# Supplementary material for: Guideline Adherence of Asymptomatic Bacteriuria Could Be Improved among General Practitioners in The Netherlands: A Survey Study
Source: Antibiotics (Basel). 2022 Jan 9;11(1):75. doi: 10.3390/antibiotics11010075 (PMC8772807; doi:10.3390/antibiotics11010075)
Supplement: Supplementary file 1 [file antibiotics-11-00075-s001.zip › antibiotics-1546004-supplementary.pdf]

antibiotics

MDPI

Supplementary Materials

Table S1. Knowledge scores

Knowledge score	n (%)
2	6 (6.4)
3	24 (25.5)
4	31 (33)
5	22 (23.4)
6	11 (11.7)

N are the number of general practitioners that earned this score

Table S2. Other reported indications for performing urine tests

Other indications for performing urine tests specified	n=5
Albuminuria	2
All of the above mentioned reasons could be used to perform urine tests, but not in all cases	1
Pain in the lower abdomen	1
Confusion	1
Control test after UTI with hematuria or when there is no improvement of symptoms after antibiotic treatment for UTI	1

N are the number of general practitioners

Table S3. Other reported policies following an abnormal urine test result

Other policies following an abnormal test result specified	n=21
Advices; antibiotics are only prescribed only if the patient has severe symptoms.	1
Antibiotics when nitrite is positive.	1
Dipslide/urine culture if necessary or when in doubt.	4
Doctor's assistant consults GP, after which a medical policy is chosen (wait-and-see policy, antibiotic prescription or additional urine tests).	2
GP decides medical policy for patients in risk groups and patients with complicated UTI, for other patients a local treatment policy is followed by the doctor's assistant.	4
Medical policy depends on patient's complaints/symptoms, medical history and comorbidity.	9
Microscopic examination when in doubt.	1
Patient in risk groups will have a consult with the GP, for other patients a local treatment policy is followed.	2

Shared decision making between the doctor's assistant and patient.	2
Urine culture when treatment with antibiotics fails.	1
We do not use urine dipsticks, only microscopic examination.	1

n are the number of general practitioners

Table S4. Other reported indications for antibiotic prescriptions

**Other indications for antibiotic prescriptions specified**	**n=11**
Combination of abnormal urine test results and the patient's symptoms	9
Depends on the urine culture result	1
Patients who are ill or have severe complaints and pain medication does not suffice	1
Patients with kidney disease or pyelonephritis in medical history	1

n are the number of general practitioners

Supplemental File S1. Checklist for Reporting Results of Internet E-Surveys (CHERRIES)[39]

**Item Category**	**Checklist Item**	**Section, page number**
**Design**	Describe survey design	4.1, page 9-10.
**IRB (Institutional Review Board) approval and informed consent process**	IRB approval	Back matter, page 11.
	Informed consent	Back matter, page 11.
	Data protection	All data were anonymously collected. Contact details were stored on a secured folder, password protected, and were accessible by the involved researchers only.
**Development and pre-testing**	Development and testing	4.2, page 10.
**Recruitment process and description of the sample having access to the questionnaire**	Open survey versus closed survey	4.1, page 9-10.
	Contact mode	4.1, page 9-10.
	Advertising the survey	4.1, page 9-10.
**Survey administration**	Web/e-mail	4.1, page 9-10.
	Context	4.1, page 9-10.
	Mandatory/voluntary	4.1, page 9-10.
	Incentives	4.1, page 9-10.
	Time/date	4.1, page 9-10.
	Randomization of items or questionnaires	4.2, page 10.

	Adaptive questioning	4.2, page 10.
	Number of items	4.2, page 10.
	Number of screens	4.2, page 10.
	Completeness check	4.2, page 10.
	Review step	4.2, page 10.
**Reponse rates**	Unique site visitor	Not applicable.
	View rate (Ratio of unique survey visitors/unique site visitors)	Not applicable.
	Participation rate (Ratio of unique visitors who agreed to participate/unique first survey page visitors	Results, page 2.
	Completion rate (Ratio of users who finished the survey/users who agreed to participate)	Results, page 2.
**Preventing multiple entries from the same individual**	Cookies used	4.2, page 10.
	IP check	4.2, page 10.
	Log file analysis	4.2, page 10.
	Registration	
**Analysis**	Handling of incomplete questionnaires	4.3, page 10.
	Questionnaires submitted with an atypical timestamp	4.3, page 10.
	Statistical correction	4.3, page 10.
